# A Network of Serum Proteins Predict the Need for Systemic Immunomodulatory Therapy at Diagnosis in Noninfectious Uveitis

**DOI:** 10.1016/j.xops.2022.100175

**Published:** 2022-05-31

**Authors:** Jonas J.W. Kuiper, Fleurieke H. Verhagen, Sanne Hiddingh, Roos A.W. Wennink, Anna M. Hansen, Kerry A. Casey, Imo E. Hoefer, Saskia Haitjema, Julia Drylewicz, Mehmet Yakin, H. Nida Sen, Timothy R.D. J. Radstake, Joke H. de Boer

**Affiliations:** 1 Department of Ophthalmology, University Medical Center Utrecht, University of Utrecht, Utrecht, The Netherlands; 2 Center for Translational Immunology, University Medical Center Utrecht, University of Utrecht, Utrecht, The Netherlands; 3 Translational Science and Experimental Medicine, Research and Early Development, Respiratory and Immunology, BioPharmaceuticals R&D, AstraZeneca, Gaithersburg, Maryland; 4 Central Diagnostic Laboratory, University Medical Center Utrecht, University of Utrecht, Utrecht, The Netherlands; 5 Laboratory of Immunology, National Eye Institute, National Institutes of Health, Bethesda, Maryland

**Keywords:** Network-based medicine, Neutrophils, Noninfectious uveitis, Proteomics, Systemic immunomodulatory therapy, IMT, immunomodulatory therapy

## Abstract

**Purpose:**

Early identification of patients with noninfectious uveitis requiring steroid-sparing immunomodulatory therapy (IMT) is currently lacking in objective molecular biomarkers. We evaluated the proteomic signature of patients at the onset of disease and associated proteomic clusters with the need for IMT during the course of the disease.

**Design:**

Multicenter cohort study.

**Participants:**

Two hundred thirty treatment-free patients with active noninfectious uveitis.

**Methods:**

We used aptamer-based proteomics (n = 1305 proteins) and a bioinformatic pipeline as a molecular stratification tool to define the serum protein network of a Dutch discovery cohort (n = 78) of patients and healthy control participants and independently validated our results in another Dutch cohort (n = 111) and a United States cohort (n = 67). Multivariate Cox analysis was used to assess the relationship between the protein network and IMT use.

**Main Outcome Measures:**

Serum protein levels and use of IMT.

**Results:**

Network-based analyses revealed a tightly coexpressed serum cluster (n = 85 proteins) whose concentration was consistently low in healthy control participants (n = 26), but varied among patients with noninfectious uveitis (n = 52). Patients with high levels of the serum cluster at disease onset showed a significantly increased need for IMT during follow-up, independent of anatomic location of uveitis (hazard ratio, 3.42; 95% confidence interval, 1.22–9.5; *P* = 0.019). The enrichment of neutrophil-associated proteins in the protein cluster led to our finding that the neutrophil count could serve as a clinical proxy for this proteomic signature (correlation: *r* = 0.57, *P* = 0.006). In an independent Dutch cohort (n = 111), we confirmed that patients with relatively high neutrophil count at diagnosis (> 5.2 × 10^9^/L) had a significantly increased chance of requiring IMT during follow-up (hazard ratio, 3.2; 95% confidence interval, 1.5–6.8; *P* = 0.002). We validated these findings in a third cohort of 67 United States patients.

**Conclusions:**

A serum protein signature correlating with neutrophil levels was highly predictive for IMT use in noninfectious uveitis. We developed a routinely available tool that may serve as a novel objective biomarker to aid in clinical decision-making for noninfectious uveitis.

Noninfectious uveitis is a spectrum of severe inflammatory disorders of the inner eye with complex inflammatory etiologic origins that often cause decreased vision or, in some cases, blindness. Vision loss as a result of inflammation and its complications can be limited or reversed by early and adequate therapy.^1^ Local or systemic corticosteroid treatment is the first-line therapy for acute noninfectious uveitis, but is associated with damaging side effects (i.e., increased intraocular pressure and cataract). Therefore, steroid-sparing agents are recommended to limit morbidity in cases where long-term treatment is required or when inflammation cannot be controlled by corticosteroids alone.^2^ Immunomodulatory therapy (IMT) is effective in preventing vision loss, but because of potential adverse effects, it is typically reserved for severe, vision-threatening uveitis.^3^^,^^4^

The requirement for IMT in patients with uveitis is based on duration of uveitis and inadequate response to topical and oral corticosteroid therapy. The severity and chronicity of noninfectious uveitis is evaluated by clinical assessment and grading of inflammation in the anterior chamber and posterior segment.^1^^,^^2^ Novel molecular tools to predict IMT objectively and early on are lacking, but are much needed to help better identify those patients who are at risk of severe disease and will need IMT during the course of the disease.^5^

Recent breakthroughs in immunoassay-based multiplex proteomics allow the simultaneous and accurate quantification of hundreds of proteins across a large dynamic range,^6^^,^^7^ including DNA-based aptamer multiplex technology of Somascan.^6^ Somascan analysis in human cohorts has revealed that the circulating blood proteome is highly structured into coregulated groups of proteins, and this information can be used to assess an individual's health status or risk for common comorbidities (e.g., cardiovascular disease).^8^, ^9^, ^10^, ^11^ We hypothesized that network-based analysis of the blood proteome of patients with noninfectious uveitis could be used to identify molecular signatures that can be exploited to stratify patients who require IMT.

## Methods

### Patient Cohorts

This study was conducted in compliance with the tenets of the Declaration of Helsinki. Ethical approval was obtained from the Medical Ethical Research Committee of the University Medical Center Utrecht. All patients signed written informed consent before participation.

Serum from 54 adult patients with noninfectious uveitis (cohort 1) was collected at the Department of Ophthalmology, University Medical Center Utrecht, Utrecht, The Netherlands (Table 1). At the time of sampling, all patients demonstrated active uveitis (new onset or relapse) according to the Standardization of Uveitis Nomenclature criteria.^12^ All patients had not received systemic treatment in the 3 months before sampling (except for 1 patient who received ≤ 10 mg oral prednisolone). Serum from 26 anonymous blood donors (University Medical Center Utrecht) with no history of inflammatory eye or inflammatory systemic disease served as control participants. For replication and validation, we included data from 111 Dutch systemic treatment-free active patients with noninfectious uveitis from the Department of Ophthalmology, University Medical Center Utrecht, Utrecht, The Netherlands (cohort 2; Table 1), and 67 systemic treatment-free North American patients with noninfectious uveitis recruited at the National Eye Institute, Bethesda, Maryland (cohort 3; Table 1).

**Table 1:** Demographic and Clinical Details for the Study Cohorts.

Cohort	Healthy controls (n = 26), Netherlands	Uveitis Cohort 1 (n = 52), Netherlands	Uveitis Cohort 2 (n = 111), Netherlands	Uveitis Cohort 3 (n=67), United States
Female/male	16/10			
Mean age (SD)	41 (11)			
Anatomical location of uveitis				
Anterior (%) HLA-B27-positive Uveitis Behçet's uveitis Idiopathic uveitis		19 (37) 19	35 (31.5) 16 1 18	-
Female/male		14/5	21/14	-
Mean age (SD)		47 (16)	44 (19)	-
Intermediate (%) Idiopathic intermediate uveitis HLA-B27-positive Uveitis Sarcoid uveitis Multiple sclerosis-associated Tattoo associated		15 (29) 15	9 (8) 7 1 1	25 (37.3) 20 - 3 1 1
Female/male		10/5	5/4	14/11
Mean age (SD)		37 (12)	40 (21)	35 (18)
Posterior (%) Birdshot uveitis Behçet's uveitis Multifocal choroiditis Idiopathic uveitis Sarcoid uveitis Punctate inner choroidopathy Ampiginous choroiditis Acute idiopathic blind spot enlargement syndrome		18 (34) 18	25 (22.5) 4 1 6 9 5	15 (22.4) 5 2 6 1 1
Female/male		9/9	15/10	15/0
Mean age (SD)		52 (12)	50 (18)	51 (15)
Pan (%) HLA-B27-positive Uveitis Birdshot uveitis Behçet's uveitis Multifocal choroiditis Idiopathic uveitis Sarcoid uveitis Sympathetic ophthalmia VKH uveitis Multiple sclerosis-associated		-	42 (38) 3 2 3 3 23 5 1 2	27 (40.3) 1 1 8 3 1 12 1
Female/male		-	21/21	15/12
Mean age (SD)		-	43 (20)	41 (16)

The distribution of female and male samples and mean age (standard deviation) for the healthy controls, and the 3 cohorts is presented. The uveitis subtype is shown for each anatomical location of noninfectious uveitis.

### Somascan Proteomic Assay

Serum tubes were kept for 30 minutes at room temperature, centrifuged at 2000*g* for 10 minutes at room temperature, and stored directly at –80° C. Serum samples were analyzed by SomaLogic using the 1.3K SomaScan assay.^13^ The samples were run with the mitigation protocol at SomaLogic to control assay interference from potential anti-self-nucleic acid autoantibodies.^14^ The SomaScan dataset after hybridization control normalization, median signal normalization, and calibration is presented in adat format and was used for analysis.

### Construction of the Serum Protein Coexpression Network

Weighted gene coexpression network analysis was conducted using the WGCNA package^15^ using a soft thresholding power of 12 for signed networks and a minimal module size of 10 proteins (nearly scale-free topology, *r*^2^ > 0.9). Modules with highly similar expression profiles (correlation of eigenprotein values, ≳ 0.75) were merged.

### Survival Analysis of Neutrophil Blood Count

The neutrophil count of 111 treatment-free active uveitis patients (cohort 2) was determined in blood samples (which was carried out on the day the patients visited the clinic for a standard diagnostic workup for uveitis at the University Medical Center Utrecht) by the CELL-DYN Sapphire automated hematology analyzer (Abbott Diagnostics) obtained by the Utrecht Patient Oriented Database.^16^ We used a single measurement, except for 2 patients who underwent multiple measurements on the same day (one patient with a 2-minute interval [4.16 × 10^9^/L and 4.23 × 10^9^/L] and another patient with 3 measurements within 1.5 hours [3.03–3.19 × 10^9^/L]), which resulted in 114 samples. To prevent data from being selected subjectively (selection bias) and to ensure representative patient measurements at time of sampling (i.e., “the average is nobody”), we kept these 3 extra measurements in our analysis of 111 patients.

The neutrophil count in the systemic treatment-free North American patient cohort (n = 67; cohort 3) was determined using the Sysmex XN-3000 automated hematology analyzer (Sysmex Corporation). The cumulative hazard rates were analyzed using the coxph() function and ggforest() functions from the survival^17^ and survminer^18^ R packages (R Foundation for Statistical Computing). To determine the best split in neutrophil count, we iteratively estimated the maximum of the standardized log-rank statistics using the surv_cutpoint() function of the survminer R package with the minimal proportion of observations per group parameter minprop ranging from 0.1 to 0.49.

### Statistical Analysis

**Figure 1:**
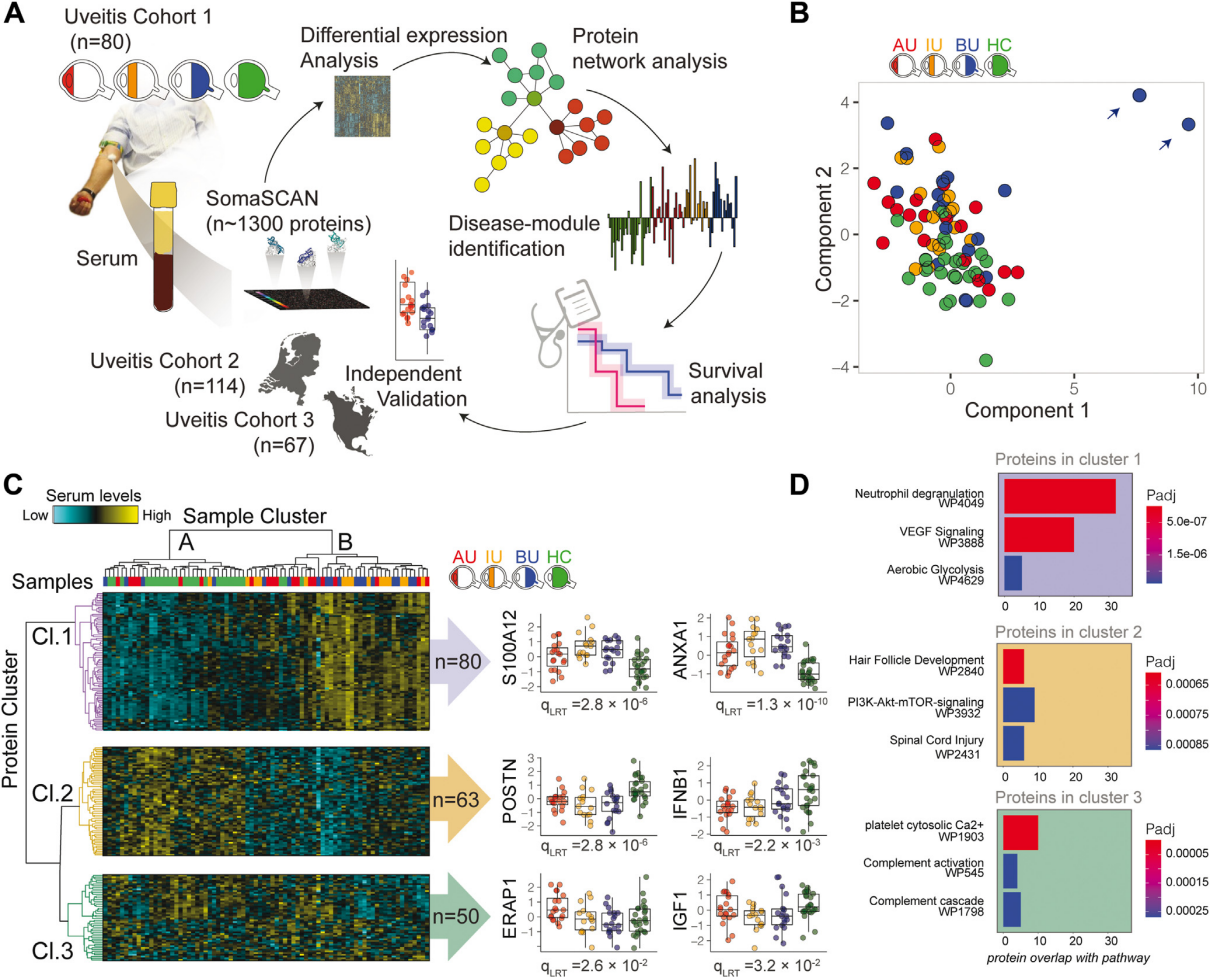
Serum proteome changes in patients with noninfectious uveitis. **A**, Schematic overview of the design of the study. **B**, Principal component analysis based on the log10 transformed relative fluorescence units of 936 detected serum proteins in 54 patients with anterior uveitis (AU), intermediate uveitis (IU), or posterior (Birdshot) uveitis (BU) and 26 healthy control participants. The blue arrows indicate 2 outlier patients with BU removed from further analysis. **C**, Hierarchical cluster analysis (using Euclidean distance with Ward’s minimum variance method) of 193 differentially expressed serum proteins (likelihood ratio test [LRT] q value, <0.05). Three overarching clusters of differentially expressed proteins (rows) are color coded. Scatterplots of representative serum proteins for each cluster are shown with their respective q values from the LRT. **D**, Top 3 enriched WikiPathways^20^ for the differentially expressed proteins in each cluster are shown, colored according to adjusted *P* value. Akt = protein kinase B; ANXA1 = annexin A1; Cl = cluster; ERAP1 = endoplasmic reticulum aminopeptidase 1; IFNB1 = interferon β1; IGF1 = insulin-like growth factor 1; mTOR = the mammalian target of rapamycin; Padj = adjusted *P* value; POSTN = periostin; S100A12 = S100 calcium-binding protein A12.

All statistical analyses were carried out in R software version 4.0.3 (2020-10-10). Very low expressed aptamers with a mean relative fluorescence unit of < 200 in all disease groups were removed, leaving 938 aptamers. Two outlier samples were identified by principal component analysis (Fig 1B) and removed. Data for 78 samples were subjected to quantile normalization using the R package preprocessCore with the function normalize.quantiles() and subsequently were subjected to Box-Cox transformation with the preProcess() function and method parameter including center, scale, BoxCox, and nzv (n = 2 aptamers removed). Differential expression analysis was conducted on 936 aptamers using a likelihood ratio function adjusting for age and sex. The qvalue R package was used for false discovery rate estimation and q <0.05 was considered statistically significant. Pathway enrichment analysis was conducted using the ClusterProfiler package^19^ and WikiPathways.^20^

### Data Availability

The full reproducible code, raw data, metadata, and Supplemental Tables 1, 2, and 3 (.xml) are available at dataverseNL via https://doi.org/10.34894/QR1VFZ.

## Results

We used SomaScan aptamer technology to measure 1305 serum proteins in 54 treatment-free patients with active uveitis (Supplemental Table 1) and 26 healthy individuals (Fig 1A). After quality control, 2 outlier samples were removed (Fig 1B). We detected 936 serum proteins, of which 193 were differentially expressed proteins between the disease and control groups (likelihood ratio test: false discovery rate, 5%; Supplemental Table 1). Global comparison by hierarchical cluster analysis clustered the samples into 2 groups; cluster A contained mostly control participants (23/26 control participants) plus 11 patients, whereas cluster B contained nearly exclusively patients (41/44 patients; Fig 1C). This analysis further discerned 3 clusters of differentially expressed proteins (C11, C12, and C13). Protein cluster C11 contained proteins that were higher in the serum of patients, including S100A12 and Annexin A1, and was enriched for the neutrophil degranulation pathway (adjusted *P* = 1.6 × 10^–21^; Fig 1D). Levels of cluster C12 proteins were generally lower in the serum of patients compared with that of control participants (e.g., interferon β1), whereas proteins of cluster C13 often showed uveitis subtype-specific expression patterns (e.g., endoplasmic reticulum aminopeptidase 1 in anterior uveitis; Fig 1C).

**Figure 2:**
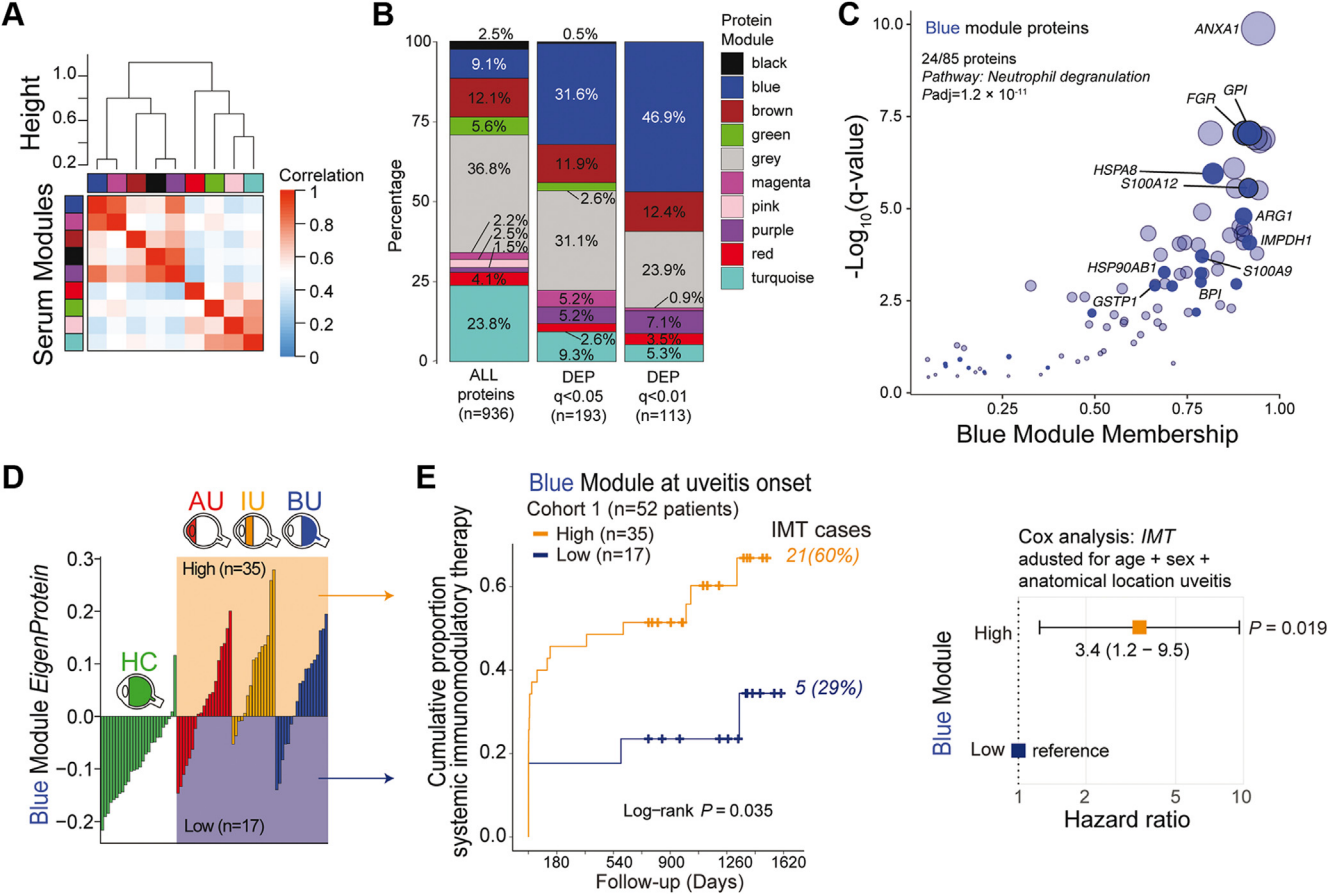
Coexpression network analysis links serum protein network to systemic immunomodulatory therapy. **A**, Weighted protein coexpression network analysis of 936 proteins distinguished 9 (color-coded) serum protein modules. The correlation of the module’s eigenprotein is color-coded from blue to red. The correlation (1 – cor[eigenproteins]) was used as a distance metric (“height” indicates the distance between clusters) for the dendrogram. **B**, Graph showing the proportion of all detected serum proteins and differentially expressed proteins (at q < 0.05 and q < 0.01) among the 9 modules identified in (**A**). Note that the grey module contains unassigned proteins. **C**, Scatterplot showing the q values from the likelihood ratio test (a measure of differential expression between patients and control participants) versus the module membership for proteins of the blue module. The size of the circles is proportional to –log_10_(q value). Twenty-four proteins (solid blue) are present in the neutrophil degranulation pathway (adjusted *P* value from enrichment analysis). **D**, Graph showing the eigenprotein value of the blue module (first principal component of the module) for control participants (green), patients with anterior uveitis (AU; red), patients with intermediate uveitis (IU; orange), or patients with Birdshot uveitis (BU; blue). Thirty-five patients showed a relatively high expression of the proteins (high group) and 17 patients displayed a relatively low expression of the proteins (low group). **E**, Cumulative event curve for the use of systemic steroid-sparing immunomodulatory therapy (IMT) in patients with high (red) or low (green) expression of the blue module as identified in (**D**). The *P* value from a log-rank test and the total IMT events during follow-up per group are shown. On the right is a corresponding forest plot (Cox proportional hazard analysis adjusted for age, sex, and anatomic location of uveitis) for the use of systemic immunomodulatory therapy among the low (reference) and high blue protein module groups. DEP = differentially expressed proteins; HC = healthy controls.

The human serum proteome functions as a biological network with structured groups of coregulated proteins.^8^ With this in mind, we constructed a coexpression network that divided the serum proteome (n = 936) into 9 highly structured protein modules (14 to 223 proteins; Fig 2A, Supplemental Table 1), with 37% of proteins falling outside of these modules (assigned to a grey module; Fig 2B). Differentially expressed proteins were overrepresented in the blue module (61/193 differentially expressed proteins in the 85-protein blue module; Fig 2B). Enrichment analysis revealed that the blue module was strongly enriched for the neutrophil degranulation pathway (adjusted *P* = 1.2 × 10^–11^). Neutrophil inflammatory proteins S100A12, IMPDH1, and ARG1 showed high module membership scores (> 0.9), further supporting that this module predominantly represents a neutrophil signature (Fig 2C).

The eigenprotein of the blue module (i.e., the first principal component of the expression data of this module) was consistently low in control participants, but similarly varied among patients across all 3 anatomic locations of uveitis (Fig 2D); 35 patients (67%) in total showed relatively high levels and 17 patients (33%) displayed relatively low levels of the blue module. Because the 2 groups of patients stratified by the blue module were highly comparable in age, sex, and anatomic location of disease (Supplemental Table 2), we hypothesized that this serum protein module represented systemic immune activity. Because steroid-sparing systemic IMT is often clinically indicated to control severe uveitis,^1^^,^^2^ we assessed if the blue module predicted IMT use over the course of clinical follow-up. Binary stratification of patients based on the expression of the blue module showed a significant difference in the probability of the 2 groups to initiate IMT treatment during follow-up (*P* = 0.035, log rank test; Fig 2E). Using a multivariate Cox analysis adjusting for age, sex, and anatomic location of uveitis, we identified that patients with a relatively high expression of the blue module at baseline significantly more often required IMT (hazard ratio, 3.42; 95% confidence interval, 1.22–9.5; *P* = 0.019; Fig 2E, Supplemental Fig 1). We conclude that the relative levels of a network of 85 serum proteins at disease onset can distinguish patients with a differential probability for requiring systemic IMT during follow-up.

**Figure 3:**
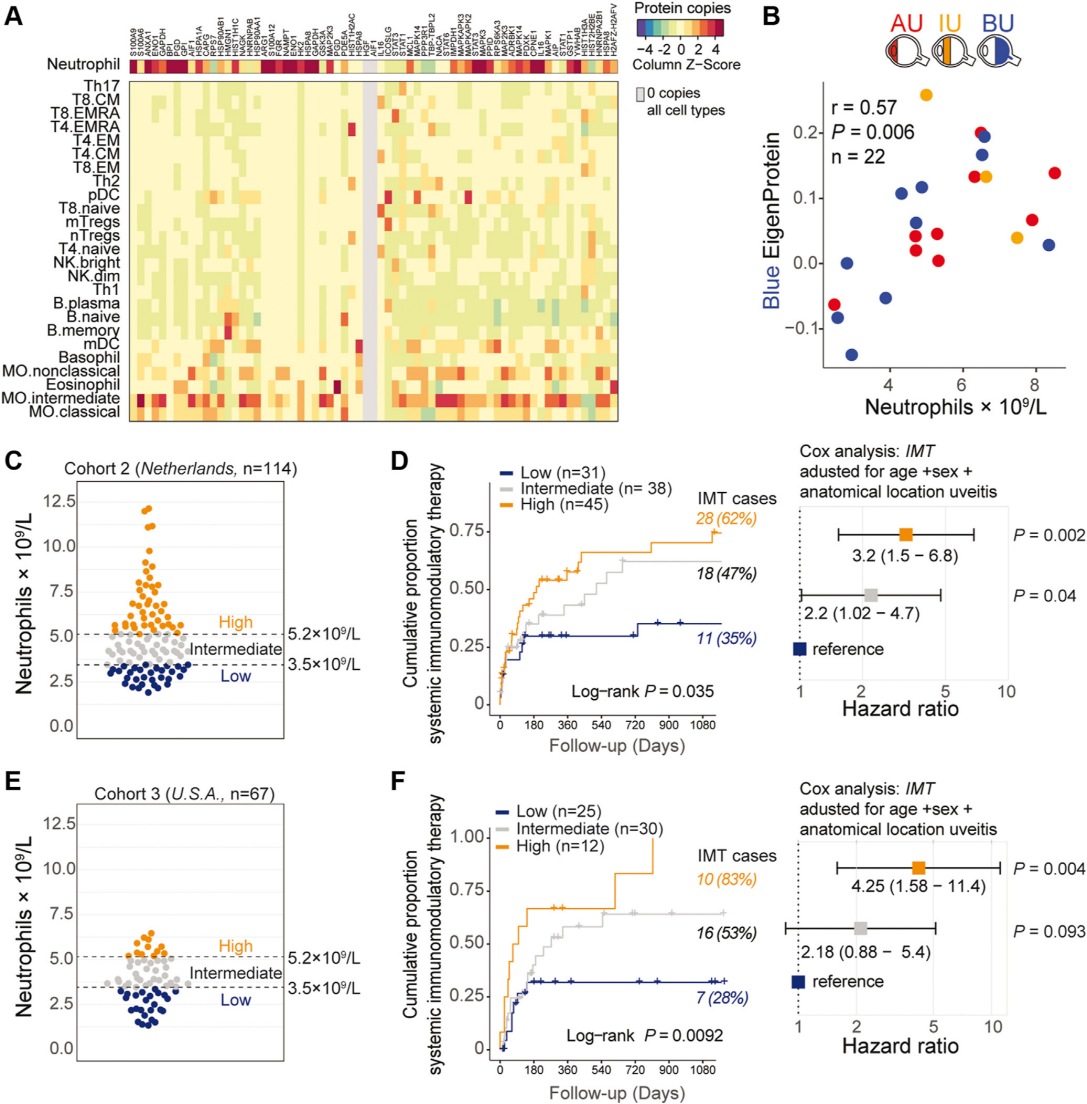
Blood neutrophil count at disease onset is a proxy for the serum signature and predicts the relative requirement for systemic immunomodulatory therapy (IMT) during follow-up. **A**, Heatmap of the mean protein copy numbers (*Z* score) in primary neutrophils and other immune cell subsets (data from Rieckmann et al^21^) for the proteins identified in the blue serum protein module. Details on the protein copies per cell type are outlined in Supplemental Table 3. AIF1, interleukin (IL)-16, MAPK14, PGD, STAT1, STAT3, HSPA8, GADPH, and EN01 have > 1 protein isoform (Supplemental Table 3). **B**, Scatterplot of the eigenprotein values for the blue module versus the blood neutrophil count for 22 patients with anterior uveitis (AU), intermediate uveitis (IU), or Birdshot uveitis (BU) with available blood neutrophil count data at uveitis onset. The correlation coefficient *r* and *P* values are from Pearson’s product-moment correlation test. **C**, Scatterplot showing the distribution of blood neutrophil counts of an independent cohort of 111 Dutch patients with noninfectious uveitis. The split points used to stratify the patients into 3 groups (low, intermediate, and high) for survival analysis are indicated. **D**, Cumulative event curve on the left showing use of systemic immunomodulatory therapy in the Dutch cohort (cohort 2; Table 1) stratified by baseline blood neutrophil group (from (**C**)). Corresponding forest plot (Cox proportional hazard analysis adjusted for age, sex, and anatomic location of uveitis) on the right for the use of systemic immunomodulatory therapy among the low (reference), intermediate, and high blood neutrophil groups. **E**, **F**, Same plots as in (**C**) and (**D**), respectively, but for a cohort of 67 systemic treatment-free United States patients with noninfectious uveitis (cohort 3; Table 1).

Given that the blue module was strongly enriched for neutrophil function (Fig 2C), we assessed whether key proteins from this module were indeed expressed in neutrophils. To this end, we compared the levels (i.e., cellular protein copies) of the 85 proteins in published proteomic data from 27 primary blood immune cell subsets.^21^ This analysis showed that for 57 proteins for which data were available in immune cells subsets, many blue module proteins, such as S100A12 and Annexin A1, were specifically highly expressed in neutrophils (Fig 3A, Supplemental Table 3). Additionally, the blue module’s eigenprotein significantly correlated with the blood neutrophil count (*r* = 0.57, *P* = 0.006; Fig 3B), suggesting that this could serve as a clinical proxy for blue module expression. Such a proxy could also overcome a major limitation of Somascan technology—the measurement of only a relative abundance of protein—and could help to define objective thresholds for the signature for independent validation.

To support the usefulness of neutrophil count as a clinical stratification tool, we sought to independently validate the association between neutrophil count and the need for IMT. In an independent cohort (cohort 2, n = 114; Table 1) of Dutch patients with uveitis who had not received IMT at the time of sampling (i.e., at diagnosis), we assessed whether blood neutrophil count was associated with the likelihood of requiring IMT over the course of follow-up. We calculated optimal split points (see “Methods”) in the blood neutrophil count that best stratified patients who did and did not require IMT, which revealed 2 major stratification points at 3.5 × 10^9^ cells/L and at 5.2 × 10^9^ cells/L (Supplemental Fig 2). We next divided patients into 3 corresponding categories of blood neutrophil groups at the time of diagnosis: low (≤ 3.5 × 10^9^/L; n = 31), intermediate (> 3.5 × 10^9^/L and ≤ 5.2 × 10^9^/L; n = 38), and high (> 5.2 × 10^9^/L; n = 45; Fig 3C) and computed hazard functions for these categories. Note that these categories all fall within the normal range for blood neutrophil count. Cox proportional hazard analysis, adjusting for age, sex, and anatomic location of uveitis, revealed a more than 3-fold higher need to start IMT for patients in the high group versus those in the low group (hazard ratio, 3.2; 95% confidence interval, 1.5–6.8; *P* = 0.002; Fig 3D, Supplemental Fig 3).

As an additional validation of the prognostic value of this association, we assessed the relationship between the proteomic signature and IMT using baseline neutrophil count as a proxy in a third cohort of 67 treatment-free patients with noninfectious uveitis (cohort 3, n = 67; Table 1) enrolled at the National Eye Institute, Bethesda, Maryland. Patients from this cohort were divided into the same categories with the same absolute boundaries as the Dutch cohort: low (≤ 3.5 × 10^9^/L; n = 25), intermediate (> 3.5 × 10^9^/L–≤ 5.2 × 10^9^/L; n = 30), and high (> 5.2 × 10^9^/L; n = 12; Fig 3E), and we assessed the association with IMT using Cox proportional hazard analysis. This analysis confirmed the significantly higher need for IMT in the high group versus the low group during follow-up (hazard ratio, 4.3; 95% confidence interval, 1.58–11.4; *P* = 0.004; Fig 3F, Supplemental Fig 4). Note that iterations of the optimal split points in neutrophil blood count from cohort 3 (measured by a different hematology analysis platform; see “Methods”) revealed optimal split points nearly identical to those of cohort 2 (3.4 × 10^9^/L and 5.2 × 10^9^/L; Supplemental Fig 2), supporting that our defined neutrophil categories are clinically robust across patient populations.

## Discussion

In this study, we showed that the levels of a serum protein network linked to blood neutrophil counts at the time of diagnosis were highly predictive of the need for IMT during follow-up. Crucially, our results revealed that standardized cutoffs in normal blood neutrophil count can serve as a routinely available proxy for the serum signature and an easy single test that could stratify patients robustly into differential risk categories for IMT.

Progress from multiple clinical studies has provided a rich armamentarium of IMT as treatment options for noninfectious uveitis.^22^, ^23^, ^24^ International treatment guidelines recommend the introduction of IMT for persistent or recurrent ocular inflammation after first-line therapy with local or systemic corticosteroids.^1^^,^^2^ However, the choice of IMT initiation also depends on severity and chronicity of the disease by clinical assessment of the anterior and posterior segment, imaging (e.g., fluorescein angiography and OCT), and the presence of systemic inflammatory disease.^2^^,^^25^ Objective biomarkers that assess disease severity and predict the need for IMT across noninfectious anatomic subtypes are sparse. Development of a general global disease activity index for uveitis still depends on an array of clinical features and is less able to predict a severe disease course in advance.^26^ The single neutrophil test proposed in this study may overcome these challenges and can capture relative disease trajectories across noninfectious subtypes at the first visit. In practice, IMT may be initiated earlier in cases of posterior uveitis resulting from the higher risk of vision loss. Indeed, our analysis confirmed that IMT use was associated with anatomic location of uveitis. Furthermore, we showed that the neutrophil serum network demonstrated strong stratification power for patients requiring IMT, even when controlling for anatomic location of uveitis, age, and sex (Supplemental Fig 1). This suggests that this molecular test may help determine early on which patients have a higher probability of requiring IMT independent of disease location and may provide an attractive new biomarker for patient stratification.

Alongside ophthalmologic assessment of disease activity, detection of the signature proxy (i.e., blood neutrophil thresholds) at disease onset can help to better identify patients who later need IMT, which is useful both for the patient in understanding the disease prognosis and for the uveitis expert in the development of treatment strategies. However, this test could also assist the general ophthalmologist in early clinical decision-making. Use of this biomarker could speed up the initiation of IMT and prevent potential undertreatment for those who ultimately will require IMT to control uveitis and prevent irreversible damage (i.e., in patients with neutrophil counts of > 5.2 × 10^9^/L at uveitis onset). Because the blood neutrophil count can be easily monitored during diagnostic workup, the implementation of our defined thresholds for prospective evaluation should be possible in most clinical settings. Importantly, these cohorts were assessed by 2 distinct, common hematology platforms for quantification of whole blood samples, suggesting that this approach is robust to technical variation across platforms used to detect neutrophil blood counts.

Because none of the 230 patients in this study were receiving systemic therapy at the time of sampling (except for 1 patient receiving low-dose prednisone in cohort 1), it is currently unknown how our findings are applicable to patients already receiving IMT, which is a limitation of our study. Additionally, longitudinal studies with repeated measurement of neutrophil blood count during periodic workup are needed to determine if this systemic immune signature is a valuable tool to complement ophthalmologic assessment of disease severity over the course of noninfectious uveitis and is able to predict if medication can be tapered safely. Additionally, it is also of interest to investigate if monitoring of neutrophil count can be used to predict disease relapse or treatment response. To this end, we envision that multiomic approaches and network-based computational analysis (combining data from transcriptomics, proteomics, etc., with mathematical modelling) will deliver the resolution and depth of information required to detect additional molecular endotypes in patients. This will help us to develop more accurate stratification tools to predict risk for complications of uveitis and treatment response to various categories of conventional and biological IMT. Similar proof-of-concept approaches for prediction of treatment response to biological therapies in rheumatology have been demonstrated.^27^

Our analysis established that the serum proteome of patients with noninfectious uveitis is highly structured and segregated in clinically relevant protein modules. In this case, we demonstrated that the blue module reflects neutrophil abundance in blood. It is interesting to speculate about whether other protein modules identified in our study may also contain clinically relevant biomarkers, but this requires further investigation. Intriguingly, the expression profile of the small magenta module correlated well with that of the blue module (Fig 2A). This is of particular interest because the magenta module comprised mostly neutrophil enzymes, such as myeloperoxidase and elastase (Supplemental Table 1), and may reflect neutrophil functions that are relevant for the pathologic features of noninfectious uveitis. Neutrophils are drivers of experimental uveitis models,^28^ and the aqueous humor of patients are infiltrated by neutrophils.^29^ Previous studies have shown that neutrophil blood count is elevated in noninfectious uveitis,^30^, ^31^, ^32^ most likely reflecting activation of systemic immune activity (i.e., inflammatory index). The S100A12 protein identified in the serum network of this study was previously correlated with disease activity in pediatric noninfectious uveitis^33^^,^^34^ and other inflammatory conditions.^35^ Therefore, the network of the large serum proteome established in this study provides a resource of potential key drivers of uveitis pathologic features and further illuminates the biological fingerprint of noninfectious uveitis.

In conclusion, we demonstrated that serum proteomics could identify a molecular signature that predicts the need for IMT in noninfectious uveitis. We could exploit the signature for the design of a simple-to-assess and widely available test as a proof of concept that omics technologies can deliver simple prognostic indicators to deliver precision care for patients with intraocular inflammatory diseases.

## Supplementary Data

Supplemantal Figure 1

Supplemantal Figure 2

Supplemantal Figure 3

Supplemantal Figure 4

Supplemantal Table 1-3
