# Supplementary material for: A Network of Serum Proteins Predict the Need for Systemic Immunomodulatory Therapy at Diagnosis in Noninfectious Uveitis
Source: Ophthalmol Sci. 2022 May 31;2(3):100175. doi: 10.1016/j.xops.2022.100175 (PMC9559086; doi:10.1016/j.xops.2022.100175)
Supplement: Supplemantal Figure 1 [file mmc1.pdf]

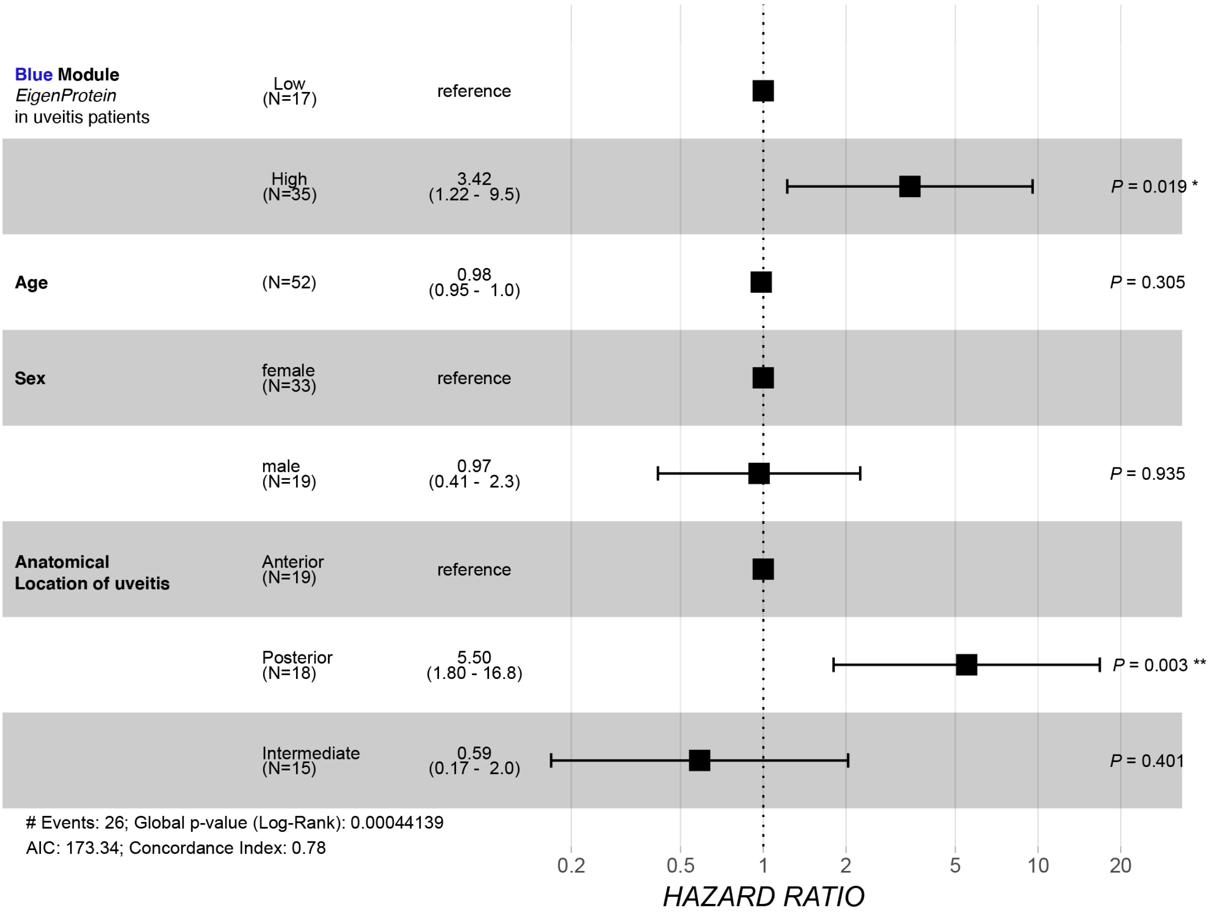

Supplemental Figure 1. Multivariate Cox model (forest plot) of the proportional hazard for patients of cohort 1 stratified by the levels of the blue module (*EigenProtein* low group versus *EigenProtein* high group). For covariates age, sex, and anatomical location of uveitis, the hazard ratio (HR) and the 95% confidence intervals of the HR are displayed.
