# Supplementary material for: A Network of Serum Proteins Predict the Need for Systemic Immunomodulatory Therapy at Diagnosis in Noninfectious Uveitis
Source: Ophthalmol Sci. 2022 May 31;2(3):100175. doi: 10.1016/j.xops.2022.100175 (PMC9559086; doi:10.1016/j.xops.2022.100175)
Supplement: Supplemantal Figure 2 [file mmc2.pdf]

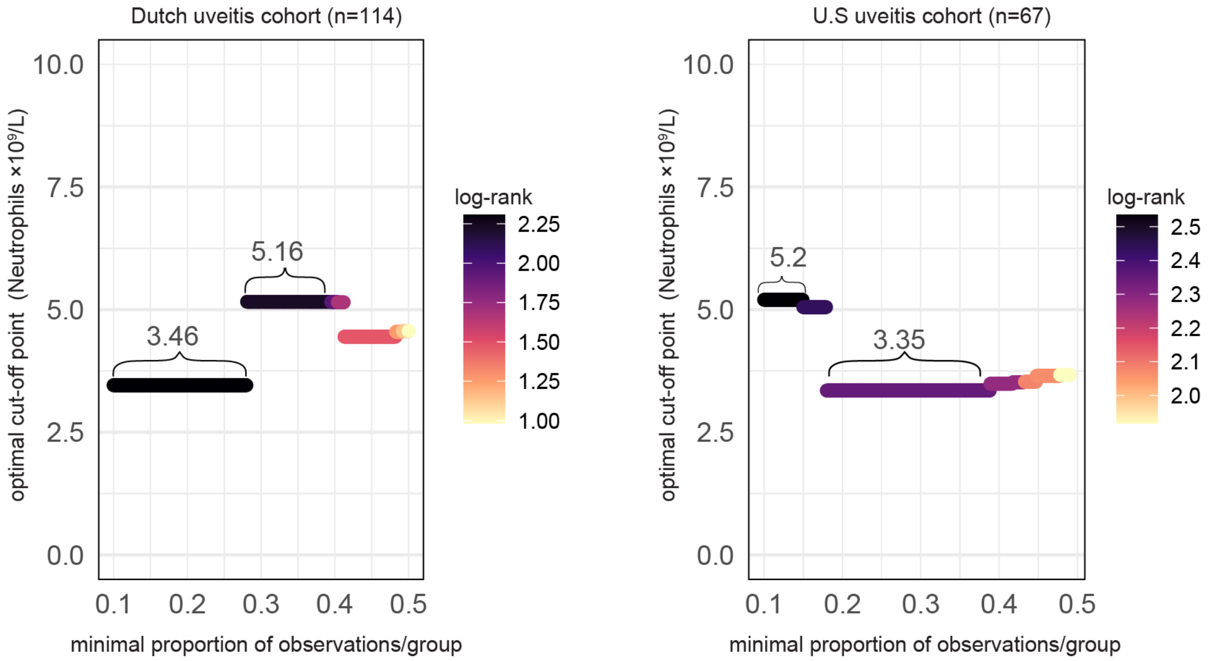

Supplemental Figure 2. The assessment of the predictive power of neutrophil blood count split points iteratively estimated from the Dutch (left) and U.S.A. (right) cohorts for immunomodulatory therapy. The split point in neutrophil count with the largest maximum of the standardized log-rank statistics is plotted for a minimal proportion of observations per group parameter *minprop* ranging from 0.1-0.49 in the *surv_cutpoint()* function of the *survminer* R package.
