# Supplementary material for: A Network of Serum Proteins Predict the Need for Systemic Immunomodulatory Therapy at Diagnosis in Noninfectious Uveitis
Source: Ophthalmol Sci. 2022 May 31;2(3):100175. doi: 10.1016/j.xops.2022.100175 (PMC9559086; doi:10.1016/j.xops.2022.100175)
Supplement: Supplemantal Figure 3 [file mmc3.pdf]

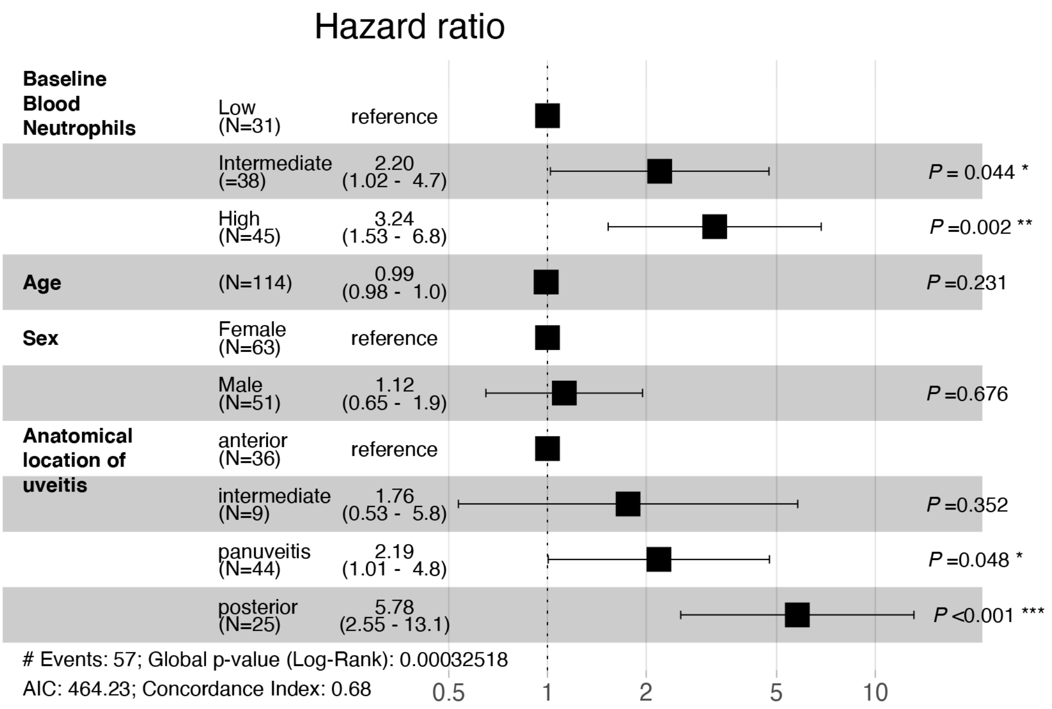

Supplemental Figure 3. Forest plot of the multivariate Cox model for systemic immunomodulatory therapy during follow-up, adjusted for age, sex, and anatomical location of uveitis for cohort 2 (n=114). Patient stratified by the baseline blood neutrophil count (proxy for the blue module): “low” (≤3.5×10^9/L, n=31), “intermediate” (>3.5×10^9/L and ≤5.2×10^9/L, n=38), and “high” (>5.2×10^9/L, n=45).
