# Supplementary material for: A Network of Serum Proteins Predict the Need for Systemic Immunomodulatory Therapy at Diagnosis in Noninfectious Uveitis
Source: Ophthalmol Sci. 2022 May 31;2(3):100175. doi: 10.1016/j.xops.2022.100175 (PMC9559086; doi:10.1016/j.xops.2022.100175)
Supplement: Supplemantal Figure 4 [file mmc4.pdf]

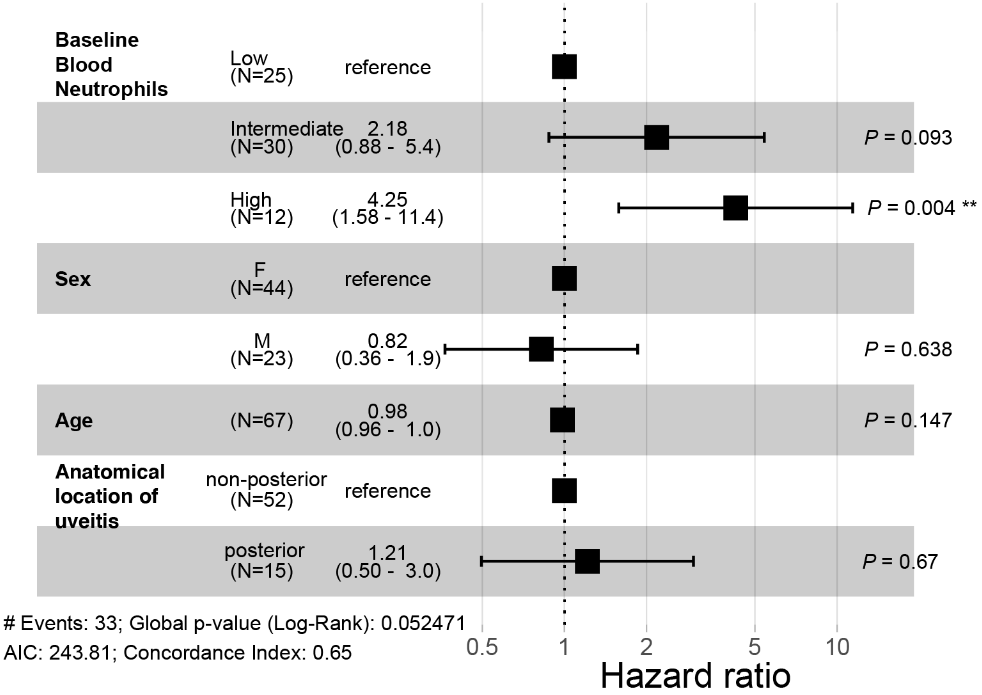

Supplemental Figure 4. Forest plot of the multivariate Cox model for systemic immunomodulatory therapy during follow-up, adjusted for age, sex, and anatomical location of uveitis for cohort 3 (n=67). Patient stratified by the baseline blood neutrophil count (proxy for the blue module): “low” (≤3.5×10^{9}/L, n=25), “intermediate” (>3.5×10^{9}/L and ≤5.2×10^{9}/L, n=30), and “high” (>5.2×10^{9}/L, n=12).
